# The Effect of Metabolic Syndrome on the Outcome of Hepatitis B-Associated Hepatocellular Carcinoma Patients After Hepatectomy: A Multicenter Study

**DOI:** 10.3389/fonc.2022.811084

**Published:** 2022-03-09

**Authors:** Junlong Dai, Xinrui Zhu, Junyi Shen, Yu Zhang, Fei Xie, Yu Yu, Kangyi Jiang, Tianfu Wen, Chuan Li

**Affiliations:** ^1^ Liver Transplantation Center, Department of Liver Surgery, West China Hospital, Chengdu, China; ^2^ Organ Transplantation Center, Sichuan Academy of Medical Sciences (Sichuan Provincial People’s Hospital), Chinese Academy of Sciences, Chengdu, China; ^3^ Department of Hepatobiliary and Pancreatic Surgery, the First People’s Hospital of Neijiang City, Neijiang, China; ^4^ Department of Hepatopancreatobiliary Surgery, the Second People’s Hospital of Yibin City, Yibin, China; ^5^ Department of Hepatobiliary Surgery, People’s Hospital of Leshan, Southwest Medical University, Leshan, China

**Keywords:** metabolic syndrome, hepatocellular carcinoma, HBV-associated HCC, hepatectomy, prognosis

## Abstract

**Background and Aims:**

With changes in dietary patterns and modern lifestyles, the prevalence of metabolic syndrome (MetS) in hepatitis B virus (HBV)-associated hepatocellular carcinoma (HCC) patients is increasing. The purpose of our study is to explore the impact of MetS on the prognosis of HBV-associated HCC patients following radical hepatectomy.

**Methods:**

Data on consecutive HCC patients who underwent radical hepatectomy were prospectively obtained and retrospectively analyzed from seven medical centers in west areas of China. Propensity score matching (PSM) analysis was conducted to balance the heterogeneity between MetS-HBV-HCC group and HBV-HCC group. Surgical outcomes have been contrasted between the two groups.

**Results:**

In 984 patients, 179 (18.19%) were diagnosed with MetS. Patients in the MetS-HBV-HCC group had higher CCI score (8.7 [0.0, 12.2] vs. 0.0 [0.0, 8.7], *p* = 0.048) and a higher rate of severe complications (Clavien–Dindo ≥3, 7.82% vs. 4.10%, *p* = 0.035), to be more precise: postoperative liver failure, hydrothorax, and hyperglycemia. Patients in the MetS-HBV-HCC group tended to have worse 5-year overall survival (OS) rate (61.45% vs. 69.94%, *p* = 0.027) and recurrence-free survival (RFS) rate (62.57% vs. 53.66%, *p* = 0.030), consistent with the results of the competing risk models. Last, MetS was identified to be an independent unfavorable prognostic factor in the multivariate analysis.

**Conclusion:**

The involvement of MetS increased the risk of postoperative complications and worsens the overall survival and recurrence-free survival time, reminding us to be more prudent to face metabolic disorder among tumor patients.

## Introduction

Metabolic syndrome (MetS) is becoming increasingly prevalent with high socioeconomic cost that has been considered a worldwide epidemic (1). MetS is a complex disorder defined by a cluster of interconnected factors including central obesity, dyslipidemia (increased triglycerides (TG) and/or reduced high-density lipoprotein cholesterol), increased fasting glucose, and increased blood pressure (2). In 1988, Reaven was the first to put forward the concept of “Syndrome X” which was later renamed as MetS (3). The International Diabetes Federation (IDF) introduced the first global standardized concept of interdisciplinary approach in 2005 (4). According to data from the China National Health and Nutrition Surveillance (2010–2012), the overall prevalence rate of MetS among Chinese adults was over 11.0%, along with an increasing incidence. Alarmingly, mounting evidence indicated that MetS was associated tightly with increased risk of cancer development and prognosis (5, 6).

Hepatocellular carcinoma (HCC) is the third leading cause of cancer-related death worldwide (7). Chronic hepatitis B virus (HBV) infection is the most common risk factor for HCC, especially in China (8). From the etiological perspective, owing to the widespread use of effective antiviral therapy, predominantly nucleos(t)ide analogs (NAs), most HCC patients with chronic HBV have achieved sustained viral control. The role of nonviral factors, such as MetS, is anticipated to be reinforced in the future.

A large number of studies had reported the discrepancy between MetS-associated HCC and hepatitis virus-associated HCC (9). However, as two independent diseases, they are not mutually exclusive, which means the coexistence would lead to a more complex clinical situation. Unfortunately, there is no study discussing that in depth. A recent epidemiological large sample size study of our team identified MetS as an independent factor associated with a 2-fold increased risk of HCC development in a population with HBV infection, suggesting a synergistic role between HBV infections and MetS (10). Considering the background of HBV-related cirrhosis, the presence of MetS could cause an inflamed liver to experience a second hit. There is an increasing strain of MetS in Asia-Pacific regions with a high prevalence of HBV-associated HCC, and the effect of superimposed MetS on HCC linked to hepatitis B accepts a growing concern.

## Patients and Methods

### Patient Selection and Study Design

**Figure 1:**
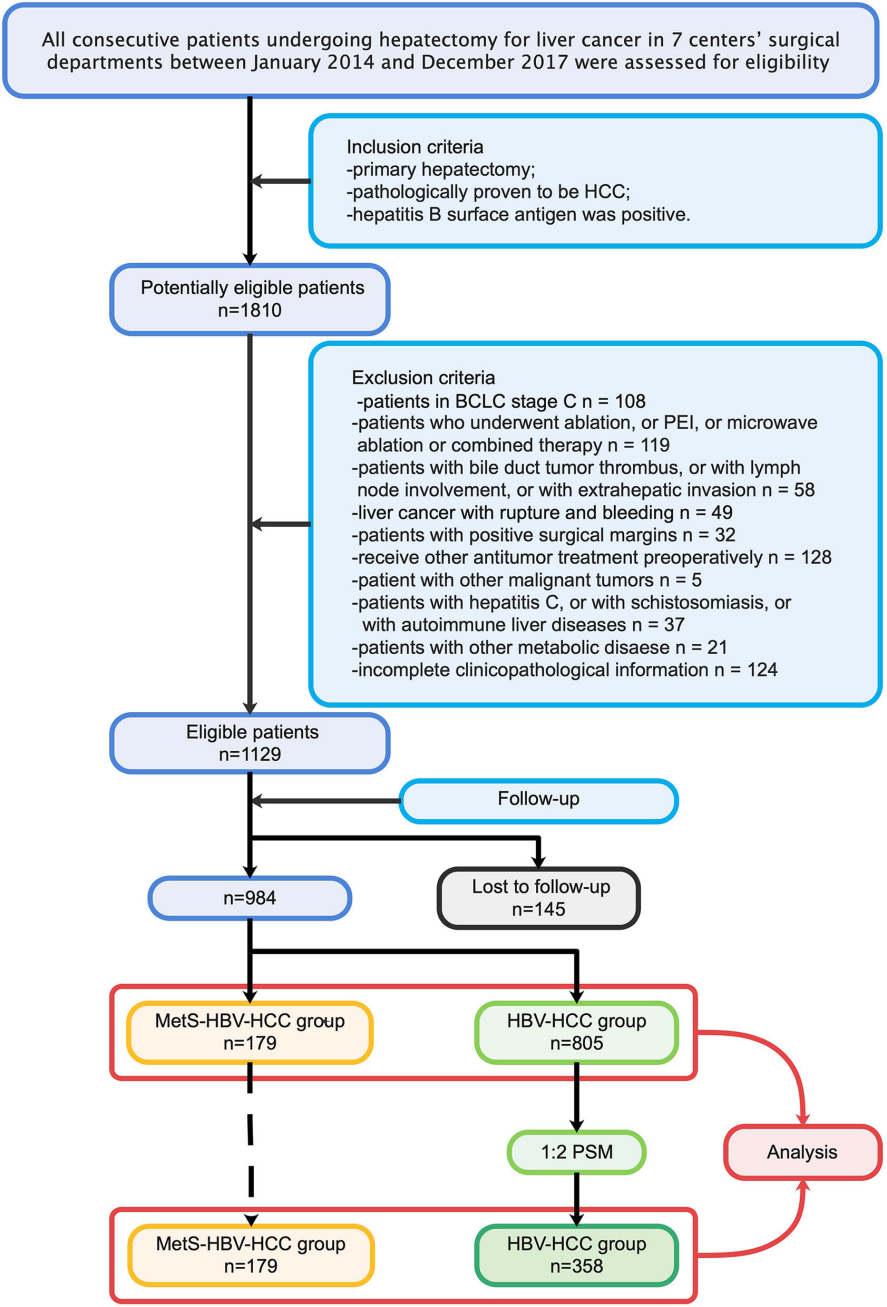
Inclusion and exclusion criteria and research design.

To ensure the quality of the study, researchers (JD, JS, YWW, WLQ, and YFC) completed data collection together and assessment independently. A total of 1,810 patients in 7 high-quality medical centers in west China were included in the candidate study population. The inclusion criteria were as follows: (1) patients who underwent primary hepatectomy; (2) patients with pathologically proven HCC; and (3) patients positive for the hepatitis B surface antibody. The exclusion criteria were as follows: (1) patients in Barcelona-Clinic Liver Cancer (BCLC) C stage; (2) patients who underwent ablation, percutaneous ethanol ablation (PEI), microwave ablation, or combined therapy; (3) patients with a bile duct tumor thrombus, lymph node involvement, or extrahepatic invasion; (4) patients with positive surgical margins; (5) patients who received other antitumor treatment preoperatively; (6) patients with other malignant tumors; (7) patients with hepatitis C, schistosomiasis, or autoimmune liver diseases; (8) patients with ruptured tumors; and (9) patients with incomplete clinicopathological information or follow-up data. Propensity score matching (PSM) was performed to adjust for other nonmetabolic factors on prognosis. The study process (in sequence) is shown in detail (**Figure 1** **)**.

The presence of pathological features was recorded and confirmed through macroscopic and histological examinations by two professional hepatic pathologists. Importantly, metabolism-related indicators were obtained before surgery and assisted by endocrinologists. The management of their metabolic syndrome was also investigated and verified by follow-up.

This study conformed to the ethical guidelines of the 1975 Declaration of Helsinki and was approved by the ethics committee of West China Hospital, Sichuan University. The requirement for informed consent was waived due to the retrospective nature of the study.

### Preoperative Assessment and Hepatectomy

All patients were diagnosed with HCC before the operation according to the American Association for the Study of Liver Diseases (AASLD) guidelines (11). Intraoperative ultrasonography (IOUS) was routinely used to confirm the status of tumors. Liver parenchyma dissection was mainly performed with an ultrasonic scalpel, a Cavitron ultrasonic aspirator (CUSA; Valleylab, Boulder, Colorado), or a water dissector (JET2; ERBE, Tübingen, Germany). All surgeries were performed by experienced liver surgeons.

### Definitions

**Figure 2:**
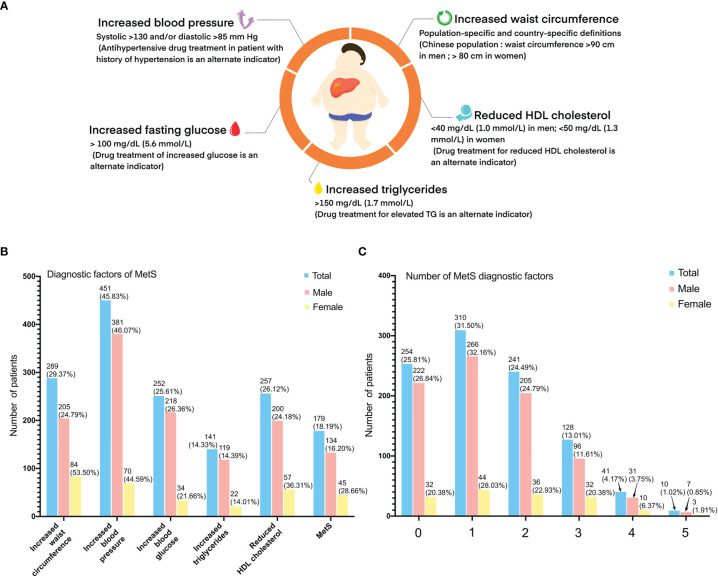
Criteria for the clinical diagnosis of metabolic syndrome: **(A)** (1) increased waist circumference (Chinese population, a man/woman >90/80 cm); (2) TG ≥150 mg/dl (1.7 mmol/L) or received drug treatment for elevated TG; (3) high-density lipoprotein (HDL) cholesterol <40 mg/dl (1.0 mmol/L) in men and <50 mg/dl (1.3 mmol/L) in women or received drug treatment for reduced HDL cholesterol levels or elevated TG; (4) systolic blood pressure ≥130 and/or diastolic blood pressure ≥85 mmHg or received drug treatment (antihypertensive drug treatment in a patient with a history of hypertension; and (5) fasting glucose >100 mg/dl (5·6 mmol/L) or received drug treatment for increased glucose levels or had been diagnosed with type 2 diabetes. **(B)** Diagnostic factors of metabolic syndrome of study population. **(C)** Number of diagnostic factors of study population.

The diagnosis of MetS was considered when at least three of the following criteria were met: (1) increased waist circumference (Chinese population, a man/woman >90/80 cm); (2) TG ≥150 mg/dl (1.7 mmol/L) or received drug treatment for elevated TG; (3) high-density lipoprotein (HDL) cholesterol <40 mg/dl (1.0 mmol/L) in men and <50 mg/dl (1.3 mmol/L) in women or received drug treatment for reduced HDL cholesterol levels or elevated TG; (4) systolic blood pressure ≥130 and/or diastolic blood pressure ≥85 mmHg or receive drug treatment (antihypertensive drug treatment in a patient with a history of hypertension); and (5) fasting glucose >100 mg/dl (5·6 mmol/L) or received drug treatment for increased glucose levels or had been diagnosed with type 2 diabetes (**Figure 2A**).

### Follow-Up

All HCC patients were regularly followed up at the first postoperative month and then every 3 months during the first postoperative 3 years and every 6 months during the subsequent years. Antiviral drugs such as entecavir or tenofovir were administered according to guidelines. Multidisciplinary teams discussed retreatment strategies upon tumor relapse. The primary endpoints were overall survival (OS) and recurrence-free survival (RFS). The survival time was defined as the interval between the date of surgery and death or the last follow-up. For patients who received liver transplantation, the date of the transplanting surgery was considered to trigger the endpoint event. The final follow-up evaluation was conducted on May 1, 2020. Recurrence and dearth situation is mentioned in **Supplementary Table S1**.

### Management

All patients with metabolic disorder were requested to the endocrine specialist clinic. Endocrinologists formulated personalized treatment plans for those patients. The therapeutic lifestyle intervention was the precondition to conducted treatment which included dietary adjustment, limiting the intake of sodium salt, increasing daily exercise, and so on. Drug therapy was conducted in patients with poor control situation. Drug using situation for metabolic disorder is mentioned in **Supplementary Table S2**.

### Statistical Analysis

Continuous variables were presented as the means ± standard deviations (M ± SD) and were compared using the Mann–Whitney *U* test. Categorical data were shown as frequencies and were compared using the *χ* ^2^ test or Fisher’s exact test. Univariate and multivariate analyses were performed using the Cox proportional hazards model. Potential risk factors with *p* < 0.05 in the univariate analysis were included in the Cox model by the step forward method. Survival was analyzed using the Kaplan–Meier method and was compared between the two groups before and after PSM using the log-rank test. The competing risk model (CRM) was used to address deaths related to tumors and deaths related to other causes, and the cumulative incidence function (CIF) was used to evaluate the true relationship between MetS and tumor-related outcomes.

PSM was adopted to overcome potential selection bias. The propensity score represents the probability of each patient being assigned to a particular condition in a study given a set of known covariables and was calculated by a logistic regression model between the two groups. The following variables possibly affected outcomes after surgery: sex, age, alpha fetoprotein (AFP) (>400 ng/ml), antivirus drugs, HBV-DNA (>10^3^ IU/ml), BCLC stage, tumor differentiation degree, microvascular invasion (MVI), satellite lesion, and albumin-bilirubin (ALBI) classification. Nearest-neighbor matching selects variables by matching a subject from the MetS-HBV-HCC group whose propensity score is closest to that of a subject from the HBV-HCC group (12). A two-sided *p*-value <0.05 was considered statistically significant. All statistical tests were performed using R version 3.5.3 (R Foundation for Statistical Computing, Vienna, Austria).

## Results

### Patient Characteristics

A total of 984 HCC patients were included in this study. Overall, 179 of the 984 patients (18.2%) had MetS (**Figures 2B, C**). There were fewer male patients in the MetS-HBV-HCC group (74.9% vs. 86.1%, *p* < 0.001), and the mean age of patients in the MetS-HBV-HCC group was higher (55.0 [46.0, 62.0] vs. 51.0 [44.0, 60.0], *p* < 0.001). As expected, the value of body mass index (BMI) (25.0 ± 3.1 vs. 22.5 ± 3.3, *p* < 0.001) in the MetS-HBV-HCC group was significantly higher than those in the HBV-HCC group. It should be noted that there are variations between the two groups in leukocytes (5.60 [4.4, 7.3] vs. 5.21 [4.2, 6.6], *p* = 0.032) and neutrophils (3.4 [2.5, 4.8] vs. 3.0 [2.3, 4.1], *p* = 0.009).

Regarding pathological characteristics, patients in the MetS-HBV-HCC group had a higher incidence rate of steatohepatitis (27.5% vs. 15.8%, *p* = 0.001). However, the proportion of cirrhotic patients was opposite (58.5% vs. 61.3%, *p* < 0.001). Detailed baseline information is listed in **Table 1**.

**Table 1:** Patient clinical characteristics.

	MetS-HCC (n = 179)	Non-MetS-HCC			
		Before PSM		After PSM	
		n = 805	*p*-value	n = 358	p-value
**Demographic data**					
Men	134 (74.9%)	693 (86.1%)	<0.001	276 (77.1%)	0.566
Age (year)	55.0 [46.0, 62.0]	51.0 [44.0, 60.0]	<0.001	55.0 [47.0, 63.0]	0.878
BMI (kg/m2)	25.0 ± 3.1	22.5 ± 3.3	<0.001	22.5 ± 3.6	<0.001
Smoking	76 (42.5%)	395 (49.1%)	0.109	155 (43.3%)	0.853
Alcohol	77 (43.0%)	310 (38.5%)	0.264	134 (37.4%)	0.211
**Liver function**					
Child–Pugh A/B	176/3	792/13	0.953	353/5	0.801
MELD score	7.27 [6.6, 8.2]	7.36 [6.6, 8.3]	0.607	7.26 [6.5, 8.1]	0.575
ALBI I/II	135/44	580/225	0.416	279/79	0.450
**Biochemical indexes**					
Total bilirubin (μmol/L)	14.2 [10.6, 18.3]	14.1 [10.9, 18.2]	0.846	14.1 [10.8, 17.7]	0.763
Albumin (g/dl)	42.3 ± 4.6	41.7 ± 4.7	0.846	42.0 ± 4.5	0.502
ALT > 50 IU/L	51 (28.5%)	223 (27.7%)	0.831	96 (26.8%)	0.681
AST > 40 IU/L	69 (38.6%)	331 (41.1%)	0.527	139 (38.8%)	0.950
**Tumor marker**					
AFP > 400 ng/ml	57 (31.8%)	283 (35.2%)	0.399	124 (34.6%)	0.519
**Virological indicator**					
HBV-DNA > 103 IU/ml	73 (48.7%)	403 (54.2%)	0.212	153 (42.7%)	0.098
Antivirus drug	86 (67.7%)	507 (76.0%)	0.049	218 (60.9%)	0.129
**Tumor staging**					
BCLC 0/A/B	22/145/12	78/684/43	0.420	29/313/16	0.140
**Hematological index**					
Erythrocyte (×1012/L)	4.6 [4.2, 5.1]	4.6 [4.2, 5.0]	0.706	4.5 [4.2, 4.9]	0.327
Hemoglobin (g/L)	144.0 [128.0, 154.0]	142.0 [130.0, 154.0]	0.889	140.0 [128.0, 152.0]	0.186
Leukocyte (×109/L)	5.6 [4.4, 7.3]	5.2 [4.2, 6.6]	0.032	5.1 [4.2, 6.3]	0.004
Platelet (×109/L)	122.0 [90.5, 168.5]	121.0 [81.0, 162.0]	0.508	118.0 [90.0, 162.5]	0.993
Prothrombin time (s)	12.2 ± 1.3	12.2 ± 1.4	0.489	12.4 ± 1.4	0.089
INR	1.1 ± 0.1	1.1 ± 0.1	0.566	1.1 ± 0.1	0.351
Neutrophil (×109/L)	3.4 [2.5, 4.8]	3.0 [2.3, 4.1]	0.009	3.0 [2.3, 3.9]	0.004
Lymphocyte (×109/L)	1.5 [1.1, 1.9]	1.5 [1.1, 1.9]	0.886	1.43 [1.1, 1.8]	0.649
NLR	2.3 [1.6, 3.3]	2.1 [1.5, 2.0]	0.051	2.0 [1.5, 2.9]	0.032
PLR	80.6 [55.6, 110.8]	85.1 [59.7, 119.1]	0.281	83.7 [60.8, 118.1]	0.468
Creatinine (μmol/L)	71.0 [60.0, 81.0]	69.0 [60.4, 79.0]	0.416	68.0 [59.0, 78.2]	0.114
**Pathology details**					
Tumor size (cm)	4.5 [3.0, 6.0]	4.4 [3.0, 6.7]	0.869	4.5 [3.0, 6.0]	0.872
Number of nodules >1	15 (8.4%)	67 (8.3%)	0.980	29 (8.1%)	0.911
Low degree of differentiation	51 (28.5%)	286 (35.5%)	0.073	113 (31.6%)	0.466
Cirrhosis	79 (58.5%)	430 (61.3%)	<0.001	184 (51.4%)	0.009
MVI	25 (15.3%)	169 (22.4%)	0.063	57 (15.9%)	0.444
Satellite nodules	10 (5.6%)	67 (8.3%)	0.218	21 (5.9%)	0.896
Steatohepatitis	49 (27.5%)	127 (15.8%)	0.001	56 (15.6%)	0.005

Shapiro–Wilk (SW) test was used to identify the normal distribution. Continuous variables satisfying normal distribution were presented as the means and standard deviations. Continuous variables not satisfying normal distribution were presented as the median and interquartile range.

HCC, hepatocellular carcinoma; MetS, metabolic syndrome; PSM, Propensity Score Match; BMI, Body Mass Index; ALT, alanine aminotransferase; AST, aspartate aminotransferase; AFP, alpha‐fetoprotein; MELD, Model for End-Stage Liver Disease; BCLC, Barcelona-Clinic Liver Cancer; NLR, neutrophil to leukocyte ratio; PLR, platelet to leukocyte ratio; INR, international normalized ratio; MVI, microvascular invasion.

### Surgical Type and Short-Term Outcomes

The similar surgical strategy resulted in a variable length of hospital stay (LOS) (13.0 [10.0, 19.0] vs. 11.0 [9.0, 15.0] days, *p* < 0.001). With respect to complications postsurgery, the incidence of the residual complications was generally higher in the MetS-HBV-HCC group than the HBV-HCC group. Similarly, patients in the MetS-HBV-HCC group had higher CCI score (8.7 [0.0, 12.2] vs. 0.0 [0.0, 8.7], *p* = 0.048) and a higher rate of severe complications (Clavien–Dindo ≥3, 7.8% vs. 4.1%, *p* = 0.035). Such disparity derived major from 3 complications in the MetS-HBV-HCC group: postoperative liver failure (3.9% vs. 1.5%, *p* = 0.034), hydrothorax (5.6% vs. 2.6%, *p* = 0.039), and hyperglycemia (27.4% vs. 10.8%, *p* < 0.001). The surgical information and short-term outcomes are reported in **Table 2**.

**Table 2:** Surgical details and short-term outcomes.

	MetS-HCC (n = 179)	Non-MetS-HCC (n = 805)	*p*-value
**Surgical procedures**			
Anatomical resection	66 (36.9%)	331 (41.1%)	0.295
Major hepatectomy	59 (33.0%)	237 (29.1%)	0.353
Laparoscopic liver resection	32 (17.9%)	126 (15.7%)	0.463
**Hospitalization information**			
LOS	13.0 [10.0, 19.0]	11.0 [9.0, 15.0]	<0.001
**Surgical complication**			
Liver failure	7 (3.9%)	12 (1.5%)	0.034
Fever	38 (21.2%)	186 (23.1%)	0.588
Hydrothorax	10 (5.6%)	21 (2.6%)	0.039
Bile leakage	14 (7.8%)	59 (7.3%)	0.820
Ascites	11 (6.2%)	26 (3.2%)	0.064
Hemorrhage	22 (12.3%)	64 (8.0%)	0.063
Parenteral nutrition	1 (0.6%)	1 (0.1%)	0.243
Hypokalemia	8 (4.5%)	31 (3.9%)	0.701
Hyperkalemia	0 (0.0%)	3 (0.4%)	0.413
Hypoglycemia	4 (2.2%)	8 (1.0%)	0.171
Hyperglycemia	49 (27.4%)	87 (10.8%)	<0.001
Wound infection	11 (6.1%)	32 (4.0%)	0.199
Pneumonia	3 (1.7%)	10 (1.2%)	0.646
Infection	4 (2.2%)	12 (1.5%)	0.477
Arrhythmia	10 (5.6%)	47 (5.8%)	0.896
Shock	5 (2.8%)	15 (1.9%)	0.425
Nausea/vomiting	8 (4.5%)	38 (4.7%)	0.885
Diarrhea	2 (1.1%)	10 (1.2%)	0.888
Constipation	7 (3.9%)	30 (3.7%)	0.907
Perioperative rescue	6 (3.4%)	14 (1.7%)	0.167
ICU	4 (2.2%)	12 (1.5%)	0.478
**Mortality**			
Mortality in 30 days	0 (0.0%)	7 (0.9%)	0.211
**Perioperative complication**			
Clavien–Dindo class I/II/III/IV/V	139/26/8/6/0	687/85/15/12/6	0.022
Postoperative severe complication	14 (7.8%)	33 (4.1%)	0.035
CCI	8.7 [0.0, 12.2]	0.0 [0.0, 8.7]	0.048

Liver failure was defined as PT > 50% and SB >50 ml/L on POD 5 (the 50–50 criteria). The Postoperative severe complication was defined as Clavein–Dindo classification ≥3. The remaining complications were defined as typical symptoms accompanied by medical intervention or invasive procedures.

HCC, hepatocellular carcinoma; MetS, metabolic syndrome; LOS, length of hospital stay; ICU, intensive care unit; CCI, comprehensive complication index.

### Survival Analysis

The median follow-up duration was 42.0 months (range of 4–60 months). In total, 311 (31.6%) patients died at the last follow-up: 69 (38.6%) in the MetS-HBV-HCC group and 217 (30.1%) in the HBV-HCC group. The component ratio of death was identical while a total of 544 (55.3%) patients with cancer recurrence have been confirmed: 112 (62.6%) in the MetS-HBV-HCC group and 432 (53.7%) in the HBV-HCC group. Remarkedly, lower rate of re-resection (12.5% vs. 24.3%, *p* = 0.007) was found in the MetS-HBV-HCC group.

**Figure 3:**
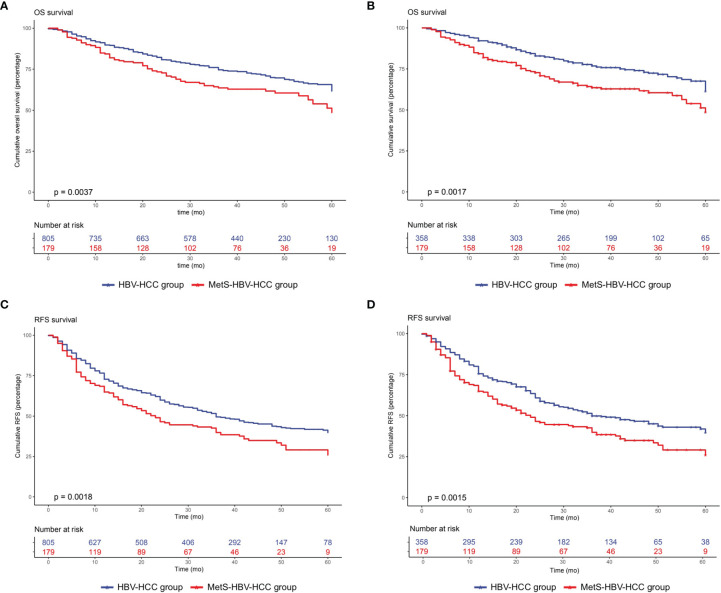
Overall survival and recurrence-free survival duration in the MetS-HBV-HCC and HBV-HCC groups: **(A)** overall survival before PSM (*p* = 0.0037) and **(B)** recurrence-free survival before PSM (*p* = 0.0018). The recurrence-free survival analysis included **(C)** overall survival after PSM at 1:2 (*p* = 0.0017) and **(D)** recurrence-free survival after PSM at 1:2 (*p* = 0.0015).

For the whole cohort, the 1-, 3-, and 5-year overall survival rates were 88.8%, 73.8%, and 68.4%, respectively. The 1-, 3-, and 5-year recurrence rates were 28.0%, 49.6%, and 55.3%, respectively. Patients without MetS had a longer OS (*p* = 0.0037, **Figure 3A**) and RFS (*p* = 0.0018, **Figure 3B**).

PSM analysis was used between the two groups in order to better monitor the confounding variables and give prominence to potential interassociation. After 1:2 PSM, 358 patients were selected to the HBV-HCC group. The patients’ baseline characteristics after PSM are also listed in **Table 1**.

**Figure 4:**
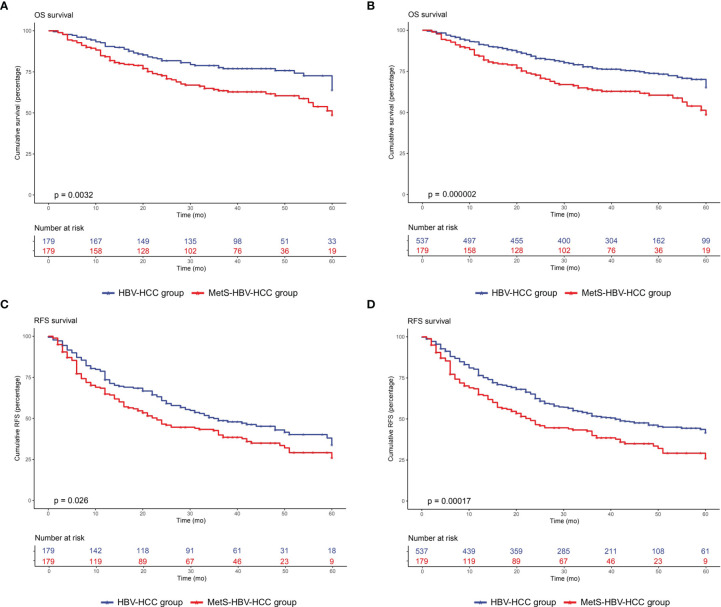
Overall survival and recurrence-free survival duration after PSM at 1:1 and 1:3 in the MetS-HBV-HCC and HBV-HCC groups: **(A)** overall survival after PSM at 1:1 (*p* = 0.0032); **(B)** recurrence-free survival after PSM at 1:2 (*p* = 0.026); **(C)** overall survival after PSM at 1:3 (*p* = 0.000002); and **(D)** recurrence-free survival after PSM at 1:3 (*p* = 0.00017).

**Figure 5:**
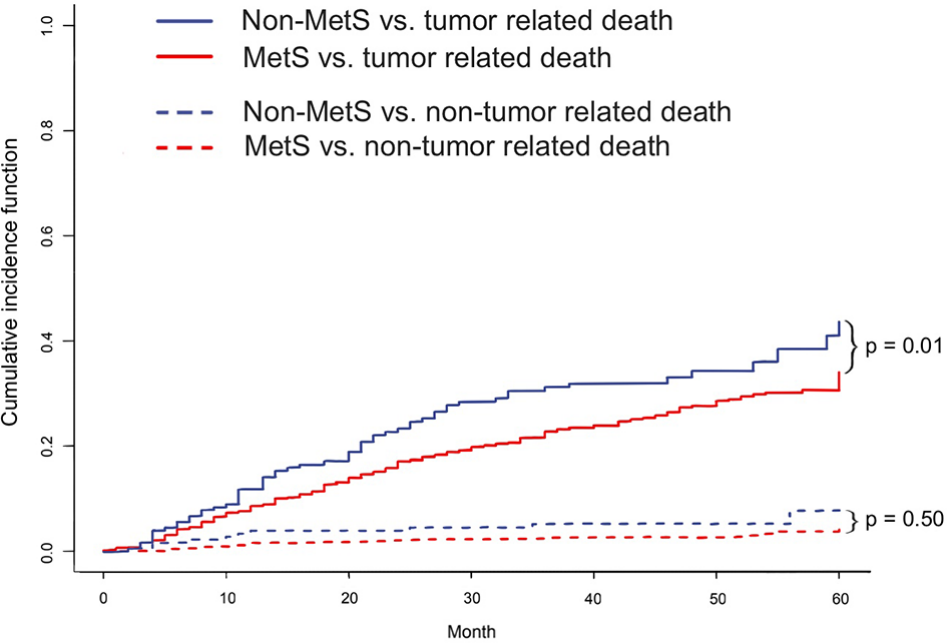
The cumulative competitive risk event incidence rate: tumor-related death (*p* = 0.01); nontumor-related death (*p* = 0.50).

After adjusting by PSM, the 5-year survival rate was 61.5% in the MetS-HBV-HCC group and 71.5% in the HBV-HCC group while the 5-year recurrence rates were 62.6% and 53.4%. In comparison, patients in the MetS-HBV-HCC group had shorter OS (*p* = 0.0017, **Figure 3C**) and RFS (*p* = 0.0015, **Figure 3D**). To verify this conclusion, PSM was also applied to investigate the impact of MetS on outcome of HBV-related HCC with match ratio, namely, 1:1 or 1:3 (**Figure 4**). The correlation between the cause of death and tumors shown by competitive risk model was linked tightly for these patients (*p* = 0.01, **Figure 5**).

### Factors Associated With Prognosis

In the univariate analysis, the presence of MetS, AFP >400 ng/ml, HBV-DNA >10^3^ IU/ml, tumor size, multiple nodules, differentiation degree, cirrhosis, MVI, satellite nodules, laparoscope, major hepatectomy, ALBI class, NLR, PLR, Clavien–Dindo class >3, and CCI were entered into the multivariate Cox model. Ultimately, the presence of MetS (*p* = 0.026), AFP >400 ng/ml (*p* = 0.008), tumor size (*p* = 0.012), multiple nodules (*p* = 0.012), MVI (*p* = 0.030), and CCI (*p* = 0.030) were considered to be independent risk factors for OS.

Next, RFS, the presence of MetS, BMI, age, HBV-DNA >10^3^ IU/ml, tumor size, multiple nodules, differentiation degree, MVI, satellite nodules, and ALBI class were entered into the multivariate Cox model. Finally, the presence of MetS (*p* = 0.025), tumor size (*p* = 0.013), multiple nodules (*p* = 0.011) MVI (*p* = 0.010), and satellite nodules (*p* = 0.008) were confirmed as independent risk factors for RFS. Independent risk factors affecting OS and RFS are listed in **Table 3**.

**Table 3:** Univariate and multivariate analyses of prognosis.

Parameter	Overall survival			Recurrence‐free survival		
	Univariate p-value	Multivariate analysis		Univariate p-value	Multivariate analysis	
		p-value	RR (95% CI)		p-value	RR (95% CI)
**Study group**						
MetS-HCC	0.002	0.026	1.557 (1.054, 2.300)	0.002	0.025	1.383 (1.024, 1.836)
BMI (kg/m^2^)	0.317			0.024	0.155	1.026 (0.990, 1.062)
**Demographic data**						
Age	0.280			0.045	0.214	0.992 (0.980, 1.005)
Male	0.134			0.191		
**Tumor marker**						
AFP > 400 ng/ml	<0.001	0.008	1.687 (1.143, 2.488)	0.254		
**Virological indicator**						
HBV-DNA > 10^3^ IU/ml	0.009	0.134	1.335 (0.915 1.947)	0.054	0.110	1.242 (0.952, 1.620)
Antiviral drug	0.280			0.417		
**Pathology details**						
Tumor size (cm)	<0.001	0.012	1.080 (1.017, 1.147)	<0.001	0.013	1.053 (1.011, 1.096)
Number of nodules > 1	0.014	0.012	2.064 (1.175, 3.624)	0.008	0.011	1.730 (1.136, 2.635)
Differentiated degree low	0.002	0.132	1.370 (0.909, 2.064)	0.020	0.331	1.152 (0.866, 1.532)
Cirrhosis	0.035	0.678	0.901 (0.487, 1.667)	0.544		
MVI	0.001	0.030	2.660 (1.099, 6.441)	0.002	0.010	2.518 (1.246, 5.088
Satellite nodules	0.012	0.542	1.268 (0.591, 2.721)	<0.001	0.008	1.964 (1.197, 3.222)
Steatohepatitis	0.359			0.248		
**Surgical procedures**						
Laparoscope	0.037	0.207	0.670 (0.360, 1.247)	0.320		
Anatomical resection	0.842			0.174		
Major hepatectomy	0.001	0.556	1.132 (0.749, 1.710)	0.137		
**Personal history**						
Smoking	0.651			0.220		
Alcohol	0.580			0.155		
**Liver function**						
Child–Pugh A/B	0.976			0.525		
MELD score	0.558			0.939		
ALBI I/II	<0.001	0.094	1.441 (0.939, 2.211)	0.055	0.713	1.451 (0.200, 10.530)
**Hematological index**						
NLR	0.007	0.681	1.008 (0.972, 1.044)	0.990		
PLR	0.002	0.862	1.000 (0.996, 1.004)	0.181		
**Surgical complications**						
Severe complications (Clavien–Dindo ≥ 3)	0.002	0.733	1.179 (0.458, 3.034)	0.533		
CCI	<0.001	0.030	1.020 (1.002, 1.039)	0.106		

CI, confidence interval; HCC, hepatocellular carcinoma; MetS, metabolic syndrome; RR, relative risk; CCI, comprehensive complication index; ALT, alanine aminotransferase; AST, aspartate aminotransferase; INR, international normalized ratio; AFP, alpha‐fetoprotein; CEA, carcinoembryonic antigen; CA19-9, carbohydrate antigen 19-9; CA125, carbohydrate antigen 125; BCLC: Barcelona-Clinic Liver Cancer stage; MVI, microvascular invasion.

## Discussion

MetS has become a global epidemic due to improvements in lifestyle and dietary patterns (13). In addition to increasing the risk of cardiovascular and cerebrovascular accidents, MetS is also closely linked to the development of malignant solid tumors such as liver cancer, colorectal cancer, prostate cancer, and breast cancer as a systemic metabolic disorder (10, 14, 15). Considering that the prevalence of MetS in HBV patients is increasing, it is rational to hypothesize the growth, especially in Asia. In patients with HBV-associated HCC who received curative hepatectomy, the downside of MetS, a possible and dangerous disease, needs to be estimated. Based on the results of our study, the presence of MetS has a negative impact on short-term and long-term survival. Previous studies indicated that MetS-HCC is associated with better long-term outcomes than viral-related HCC. It is worth noting that MetS-related patients commonly developed HCC with mild underlying liver disease or low serum AFP levels (16, 17). These conclusions, however, are the research outcome of a single factor and are not capable of representing the comprehensive situation in clinical practice. Based on the “second hit” theory, we aim to investigate the superimposed role of both adverse factors. Using different models (the CRM model, PSM model, and Cox model), we provided more convincing evidence that HBV-related HCC patients experienced worse prognosis when accompanied by MetS.

Given the effect of MetS, the major postoperative complications were higher in the MetS-HBV-HCC group. The liver is vulnerable to hepatocellular injury after resection, particularly when cirrhosis presented (18). The group of patients with viral hepatitis complicated with MetS had two types of liver disease backgrounds, which may be responsible for the high incidence of liver failure. A recent study by Wang et al. showed that MetS may accelerate the progression of liver disease in patients with chronic HBV infection and synergistically induce cirrhosis or even HCC development, demonstrating the mutually reinforcing role of MetS and viral hepatitis (19). Moreover, a more complex background of liver disease could also lead to an increased incidence of hypoproteinemia, which provides the necessary conditions for hydrothorax. Hyperglycemia is a common phenomenon resulting from postoperative stress. For the effect of metabolic abnormalities, MetS impaired the glycemic regulation after surgery (20). From another, the prolonged LOS in patients with MetS was associated with prolonged postoperative recovery, reflecting the high rates of liver infectious complications. Therefore, for patients with MetS, the preoperative assessment should be performed more strictly: the residual liver function criteria should be appraise more accurately and the perioperative management should receive more attention.

In terms of long-term survival, the presence of MetS had a negative impact on patients. This might be associated with higher levels of inflammation, insulin resistance, and specific tumor biological behaviors.

We found that patients with MetS had higher serum leukocyte and neutrophil counts, suggesting higher systemic inflammation levels, consisting with previous reports (21). A high level of systemic immune inflammation often presents a negative correlation with the prognosis of HCC (22). Windt et al. reported that neutrophil extracellular traps (NETs) formed after neutrophil apoptosis promoted the inflammatory process of NAFLD and the development of HCC, which revealed a potential association between neutrophils, MetS, and HCC (23). Increased inflammation leads to tumor recurrence and metastasis. In support of this notion, the inflammatory response is consistent with the pathophysiology of NAFLD, and when combined with viral hepatitis, it can form an inflamed tumor environment and ultimately worsen the prognosis (19).

Insulin resistance (IR), as a core mechanism of MetS, also plays an important role in HCC (2). Kim et al. demonstrated that HBV-related HCC was related to the dysregulation of insulin-IGF-1/IGFBP function and that the overexpression of IGF2 accelerated the formation of liver tumors with the hepatic expression of MYC and AKT1. In addition to the occurrence, IR also affects HCC recurrence. HCC patients with IR experience an aggressive tumor biology, worsening the prognosis of HCC patients with MetS (24, 25). Moreover, IR is also related to drug resistance in HCC (26). In summary, IR from MetS complicated with hepatitis B poses a complicated inflamed liver environment for HCC and leads to a relatively poor treatment outcome.

Patients with MetS presented a higher recurrence rate, indicating worse biological behavior. The proportion of patients with MetS who underwent re-resection was also lower, demonstrating the worse systemic or liver conditions during tumor recurrence. Active tumor cell lipid synthesis is a characteristic of high invasiveness (27). Highly active glycolysis and glutamine metabolism could also promote tumor metastasis and might provide a theoretical basis for an aggressive tumor biology in patients with MetS (28). Besides the presence of MetS, tumor size, AFP, ALBI, and CCI were well-established prognostic factors of HBV-related HCC. Therefore, our results were consistent with those from numerous previous studies.

However, there are also two diseases at the same time which could give rise to the death of such patients. To elucidate that the MetS propel the development of HCC, risk competing model was applied. From the result of the risk analysis, tumor was the major factor driving the occurrence of death instead of the nontumor factor, which means metabolic complications, like cardiovascular disease, diabetic nephropathy, or apoplexy. It strengthened reliability of the conclusion and clarified that the negative impact of MetS mainly focus on the tumor and liver for patients with HCC rather than other organs or systems (29).

### Limitations

Our study has several limitations. First, this was a retrospective study, selection bias cannot be ruled out. However, we adopted several models to achieve a consistent conclusion. Second, we did not consider the possible change in the MetS status. The effect of MetS might have been underestimated. The sample size was relatively large, and patient data were obtained from multiple centers. Thus, the results still provide some insight into the effect of MetS on HCC. Third, there may have been some inconsistency given that all patients might not have chosen the same hospital for continuous follow-up. Lastly, the treatment of MetS and hepatitis B was not fully considered; therefore, the effect of treatment regimen on prognosis was less discussed.

## Conclusion

In the HBV endemic, we provided convincing evidence that HBV-related HCC patients with MetS had worse prognosis after hepatectomy. Liver surgeons should consider the challenge of MetS when formatting surgical strategy.

## Data Availability Statement

The raw data supporting the conclusions of this article will be made available by the authors, without undue reservation.

## Ethics Statement

The studies involving human participants were reviewed and approved by Ethics Committee on Biomedical Research, West China Hospital of Sichuan University. The patients/participants provided their written informed consent to participate in this study.

## Author Contributions

All authors’ contribution was summarized into the following category:

I. Conception and design: JD, XZ, JS, CL, and TW

II. Administrative support: CL and TW

III. Provision of study materials or patients: JD, XZ, JS, CL, and TW

IV. Collection and assembly of data: JD, XZ, JS, YZ, FX, YY, and KJ

V. Data analysis and interpretation: JD, XZ, and JS

VI. Manuscript writing: all authors

VI. Final approval of manuscript: all authors

All authors listed have made a substantial, direct, and intellectual contribution to the work and approved it for publication.

## Funding

This study was supported by grants from the Health and Family Planning Commission of Sichuan Province (17PJ393), the National Natural Science Foundation of China (82070625, 81900576), the Science and Technological Supports Project of Sichuan Province (2018SZ0204, 2019YJ0149), the Science and Technology Project of Chengdu (2018-YF05-01460-SN), the State Key Scientific and Technological Research Programs (2017ZX10203207-003-0020), and the Post-Doctor Research Project, West China Hospital, Sichuan University (2020HXBH069).

## Conflict of Interest

The authors declare that the research was conducted in the absence of any commercial or financial relationships that could be construed as a potential conflict of interest.

## Publisher’s Note

All claims expressed in this article are solely those of the authors and do not necessarily represent those of their affiliated organizations, or those of the publisher, the editors and the reviewers. Any product that may be evaluated in this article, or claim that may be made by its manufacturer, is not guaranteed or endorsed by the publisher.

## Supplementary Material

Supplementary Table 1 Information on death and recurrence. HCC, hepatocellular carcinoma; MetS, metabolic syndrome; TACE, transcatheter arterial chemoembolization; Ablation therapy: radiofrequency ablation or microwave ablation. The recurrence information included 1033 patients, and patients with an unknown recurrence status (n=105) were excluded.

Supplementary Table 2 Management of metabolic syndrome. HCC, hepatocellular carcinoma; MetS, metabolic syndrome.
