# Supplementary material for: The Effect of Metabolic Syndrome on the Outcome of Hepatitis B-Associated Hepatocellular Carcinoma Patients After Hepatectomy: A Multicenter Study
Source: Front Oncol. 2022 Mar 9;12:811084. doi: 10.3389/fonc.2022.811084 (PMC8959675; doi:10.3389/fonc.2022.811084)
Supplement: Supplementary Table 1 — Information on death and recurrence. HCC, hepatocellular carcinoma; MetS, metabolic syndrome; TACE, transcatheter arterial chemoembolization; Ablation therapy: radiofrequency ablation or microwave ablation. The recurrence information included 1033 patients, and patients with an unknown recurrence status (n=105) were excluded. [file Table_1.docx]

Supplementary Tables

| Supplementary Table 1︱**Information on death and recurrence** | | | |
| --- | --- | --- | --- |
|  | MetS - HBV‐HCC  n = 179 | HBV‐HCC  n = 805 | P value |
|  |  |  |  |
| **Classification of cause of death** | | | |
| Number of deaths | 69 (38.6%) | 242 (30.1%) | 0.027 |
| Cause of death |  |  |  |
| Tumor-related death | 59 (85.5%) | 217 (89.7%) | 0.712 |
| Liver failure | 4 (6.8%) | 9 (4.2%) |  |
| Cardiovascular and cerebrovascular disease-related death | 1 (1.7%) | 5 (2.3%) |  |
| Other reason | 0 (0.0%) | 1 (0.5%) |  |
| Unknown | 5 (8.5%) | 10 (4.6%) |  |
| **Recurrence and treatment** | | | |
| Number of recurrences | 112 (62.6%) | 432 (53.7%) | 0.030 |
| Treatment for recurrence | | | |
| Re-resection | 14 (12.5%) | 105 (24.3%) | 0.007 |
| Liver transplantation | 3 (2.7%) | 17 (3.9%) | 0.529 |
| TACE | 44 (24.6%) | 166 (38.4%) | 0.868 |
| Ablation therapy | 1 (0.6%) | 19 (4.4%) | 0.079 |
| Immune or targeted drugs | 3 (1.7%) | 14 (3.2%) | 0.761 |
| Chemoradiotherapy | 4 (2.2%) | 24 (5.6%) | 0.397 |
| **Abbreviations**: HCC, hepatocellular carcinoma; MetS, metabolic syndrome; TACE, transcatheter arterial chemoembolization; Ablation therapy: radiofrequency ablation or microwave ablation. The recurrence information included 1033 patients, and patients with an unknown recurrence status (n=105) were excluded. | | | |

| Supplementary Table 2**︱Management of metabolic syndrome** | | |
| --- | --- | --- |
|  | MetS - HBV‐HCC  n = 179 | HBV‐HCC  n = 805 |
|  |  |  |
| **Increased blood glucose** |  |  |
| Number of patients | 105 (58.7%) | 147 (18.3%) |
| Insulin injection | 30 (38.6%) | 37 (25.2%) |
| Metformin | 8 (7.6%) | 6 (4.1%) |
| Other oral drugs | 2 (1.1%) | 1 (0.1%) |
| **Increased blood pressure** |  |  |
| Number of patients | 147 (82.1%) | 304 (37.8%) |
| Oral drugs | 47 (32.0%) | 70 (23.0%) |
| Aspirin | 2 (1.4%) | 11 (3.6%) |
| **Increased triglycerides** |  |  |
| Number of patients | 87 (48.6%) | 54 (6.7%) |
| Statins drugs | 2 (2.3%) | 3 (5.6%) |
| **Abbreviations**: HCC, hepatocellular carcinoma; MetS, metabolic syndrome. | | |
